# Bioaccumulation Rate of Non-Biodegradable Polystyrene Microplastics in Human Epithelial Cell Lines

**DOI:** 10.3390/ijms252011101

**Published:** 2024-10-16

**Authors:** Ilaria Conti, Cinzia Brenna, Angelina Passaro, Luca Maria Neri

**Affiliations:** 1 Department of Translational Medicine, University of Ferrara, 44121 Ferrara, Italy; ilaria.conti@unife.it (I.C.); cinzia.brenna@unife.it (C.B.); angelina.passaro@unife.it (A.P.); 2 Laboratory for Technologies of Advanced Therapies “LTTA”—Electron Microscopy Center, University of Ferrara, 44121 Ferrara, Italy

**Keywords:** microplastic, polystyrene, bioaccumulation, release, cell survival

## Abstract

Environment plastic accumulation has been attracting the attention of both political and scientific communities, who wish to reduce global pollution. Plastic items have been detected everywhere, from oceans to the air, raising concerns about the fate of plastics within organisms. Leaked plastics are ingested by animals, entering the food chain and eventually reaching humans. Although a lot of studies focused on the evaluation of plastic particles in the environment and living organisms have already been published, the behavior of plastic at the cellular level is still missing. Here, we analyzed the bioaccumulation and extrusion trend of two differently sized plastic particles (1 and 2 µm), testing them on three human epithelial cell lines (liver, lung, and gut) that represent epithelial sites mainly exposed to plastic. A different behavior was detected, and the major plastic uptake was shown by liver cells, where the 1 µm beads accumulated with a dose-dependent profile. Moreover, a 60% reduction in the content of 1 µm particles in cells was evaluated after plastic removal. Finally, the viability and proliferation of the three human cell lines were not significantly affected by both the 1 and 2 µm beads, suggesting that cells might have a defense mechanism against plastic exposure risk.

## 1. Introduction 

The ubiquitous leakage of plastic objects into the environment is raising global awareness about regulations on plastic production and usage, as well as alternative strategies (e.g., recycling and use of plastic alternatives) to reduce the ecosystem’s pollution [1]. Although no international official consensus has been reached regarding the definition of microplastics (e.g., size cutoff and materials involved), it is possible to define microplastics (MPs) as synthetic, high-molecular-weight compounds and powdered plastic particles smaller than 5 mm in size [2,3]. Moreover, MPs are classified as primary and secondary ones [2]. These latter contain plastic waste, fibers, or waste products. They may be continuously generated by biotic and abiotic degradation of larger plastic litter. Moreover, once formed, due also to a low biodegradation rate, they mostly remain in the environment, contaminating marine and air sources [2]. It has been estimated that more than 75% of MPs enter the ocean from terrestrial sources. Refs. [4,5,6,7], provoking a cascade effect, where animals, fishes, plants, and, consequently, the human body, which is the final consumer in the food chain, are involved [2,8,9,10,11]. As demonstrated by their detection in foodstuffs (e.g., sugar, honey, beer, and drinking water), MPs contamination negatively influences the whole population’s health [12,13,14,15]. This impact is increased if we consider not only all the waste products generated during plastic polymerization reactions (e.g., plasticizers, catalysts, initiators, and solvents, as well as diverse plastic polymer types) but also the environmental pollutants adsorbed by MPs, such as antibiotics and pesticides (e.g., dichlorodiphenyltrichloroethane, polycyclic aromatic hydrocarbons, and polychlorinated biphenyl) [10,11,16]. Altogether, these phenomena amplify MPs potential hazardousness and environmental toxicity [17,18]. However, although the fate of MPs has not been fully clarified yet, inhalation and food consumption have been established as the two main routes of MPs intake in humans [19]. Among these two, ingestion has been considered the major route of MPs exposure [20]. In fact, it has been demonstrated the MPs exposure might occur via ingestion, even for those particles that are commonly found in the air [20,21]. MPs can be inhaled by fibers, aerosols, and fertilizers, as well as ingested from food, drinking water, and beverages [2]. Plastic debris has been found in human placenta, stool, and lungs [22,23,24]. In vitro and in vivo experiments have shown a dependency between nanoparticle accumulation and exposure and occurrence of inflammation: the most described MPs effects relate to human digestive, respiratory, endocrine, reproductive, and immune systems [2]. The main symptoms of this contamination include increased reactive oxygen species production, lipid metabolism disturbances, gut microbiota dysbiosis, and neurotoxicity [25,26,27,28,29]. It has also been demonstrated that MPs can alter the intestinal microbiome, leading to various gastrointestinal symptoms, such as abdominal pain and bloating, as well as nausea and vomiting when ingested orally, because of the presence of environmental toxins [30,31]. When inhaled, MPs can cause oxidative stress in the airways and lungs, causing tissue inflammation and damage, low blood oxygen concentration, and different respiratory symptoms such as coughing, sneezing, and shortness of breath, which can increase the risk of chronic obstructive pulmonary disease [28]. In vitro studies on human lung cells have demonstrated that MPs accumulation promotes the expression of genes and proteins, involving the activation of innate immunity [19,29]. Moreover, MPs also alter the endocrine system, interfering with the production, release, transport, metabolism, and elimination of hormones, causing endocrine disorders, including metabolic, developmental, and even reproductive disorders (i.e., infertility and congenital malformations) [32]. Although the correlation between MPs and phenomena such as inflammation and ROS levels increasing looks clear, there is still a gap to be filled in [33,34]. There are still a few studies regarding the possible long-term effects on human health. Domenechand and co-workers proposed an in vitro study with Caco-2 cells, which were exposed to 0.26 µg/cm^2^ of polystyrene (PS) nanoplastics for 8 weeks, to mimic a long exposure time, finding genotoxicity and increased levels of ROS [35]. However, despite these results, at present, a lot of information is still missing on MPs’ behavior at the cellular level, and this is often controversial. Although most available data are related to particles with dimensions above 10 or 50 µm, less data have been described for smaller particles [36]. Potential cellular toxicity of plastics was hypothesized by molecular dynamic simulations, to understand whether plastic features (i.e., sizes, concentrations, and polymer types) could somehow be related to cellular uptake [37]. Due to their hydrophobicity, plastic polymer chains could change the lipid bilayer structure of the cell membrane, altering their biological functions and promoting cell death [38]. From this point, we aimed to understand small PS beads’ behavior on three human epithelial cell lines that represent the main human districts involved in plastic contamination: Mahlavu (hepatocellular carcinoma), A549 (lung carcinoma), and HCT-116 (colorectal carcinoma). Our central hypothesis was to define a direct correlation between small particles, density, and exposure time. Therefore, we wanted to analyze at a single cell level how bead size (1 µm and 2 µm), concentration, and exposure time might influence both the internalization and potential cytotoxicity. 

## 2. Results

### 2.1. Characterization of Polystyrene Microplastics (PS-MPs)

**Figure 1:**
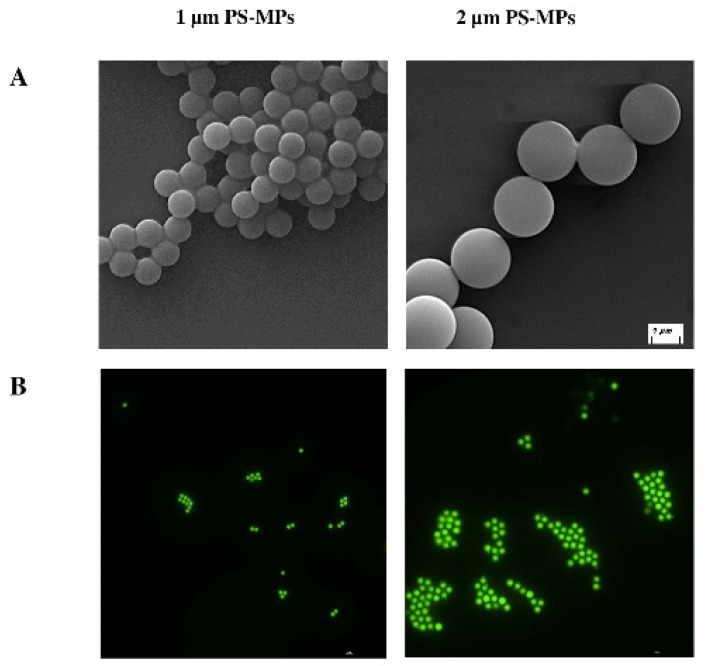
PS-MPs microscopy characterization. (**A**) SEM images of 1 and 2 µm ulPS-MPs, also representative of the corresponding flPS-MPs (scale bar: 1 µm); (**B**) fluorescent microscopy images of 1 and 2 µm flPS-MPs excited at 488 nm (scale bar: 10 µm).

For this study, two typologies of MP beads were used: unlabeled polystyrene (ulPS-MPs) and fluorescent labeled polystyrene (flPS-MPs), with 1 and 2 µm of diameter. To assess the morphology, dimensions, and fluorescent characterization and exclude false positives (i.e., presence of small debris that could be released from MPs) from both flPS-MP and ulPS-MPs, scanning electron (SEM) and fluorescent microscopies were used. Figure 1 shows PS-MPs images acquired by SEM (Figure 1A) and fluorescent microscopy (Figure 1B). SEM characterization shows uniform beads, ranging from 1 ± 0.05 µm to 2 ± 0.1 µm, respectively, in accordance with the manufacturer. Fluorescent microscopy shows PS-MPs displaying green color emission when excited at 488 nm. 

### 2.2. Internalization of 1 µm PS-MPs by Mahlavu Liver Cells

As the experimental setup consisted in exposing cells to PS-MPs, and to obtain the desired number of particles to treat the cells, we first converted the number of PS-MPs per well (mm^2^) into µg of polystyrene (Supplementary Material, Table S1). 

To understand whether Mahlavu liver cells could internalize PS-MPs, we executed a plethora of experiments, where cells were treated with different densities (Supplementary Material, Table S2) of beads at two distinct time points (24 and 48 h). 

**Figure 2:**
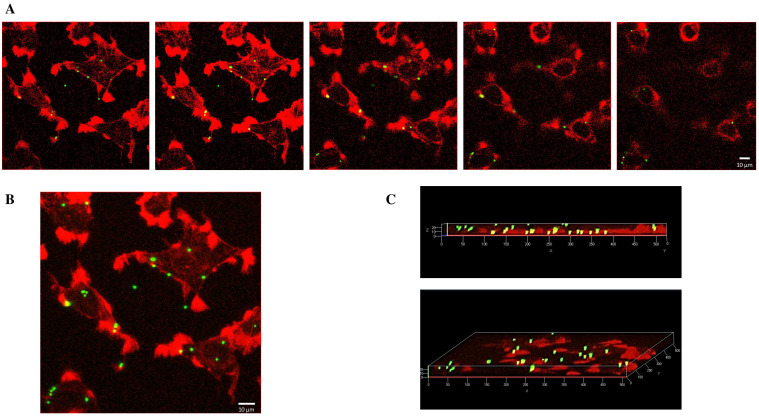
Confocal microscopy of Mahlavu cells after 24 h of exposure to 1 µm PS beads (5000 beads/mm^2^). (**A**) Five consecutive optical sections, Z-step: 0.3 µm; (**B**) Z-stack projection deriving from the superimposition of 29 optical sections taken 0.3 µm apart; (**C**) 3D views of the Z-stack along the x-axis (upper image) and the z-axis (lower image). Cytoskeleton: red (Phalloidin Alexa Fluor-555 conjugated); PS-MPs: green/yellow. Scale bar: 10 µm.

Figure 2 shows a confocal analysis of Mahlavu cells, after administration of 5000 beads/mm^2^ for 24 h. Single optical sections reveal PS-MP beads located in different areas and spatial levels within the cells (Figure 2A), whereas the superimposition of single images into a Z-stack projection allowed for the detection of four to six beads per cell (Figure 2B). Additionally, a 3D view illustrated the spatial distribution of beads inside the cells, where a specific localization pattern, including cytoskeleton extensions and a juxtanuclear region, was also observed (Figure 2C).

**Figure 3:**
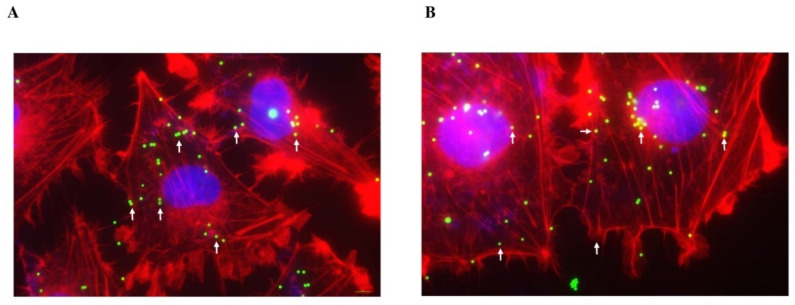
Fluorescent microscopy of bioaccumulation and subcellular localization of 1 µm flPS-MPs in Mahlavu cells after 48 h of exposure time (20,000 beads/mm^2^). The image in (**A**) was acquired with a Plan-Apochromat 60×/1.45 in oil objective, whereas the one in (**B**) was acquired with a Plan-Apochromat 100×/1.45 in oil objective, to zoom in and obtain a more detailed visualization; localization of the PS-MPs within cells is denoted by white arrows. Nucleus: blue (DAPI); cytoskeleton: red (Phalloidin Alexa Fluor-555 conjugated); PS-MPs: green/yellow. Scale bar: 10 µm.

Figure 3A depicts the bead bioaccumulation and localization along the cytoplasm in Mahlavu cells after 48 h of plastic exposure. A more magnified visualization is shown in Figure 3B.

**Figure 4:**
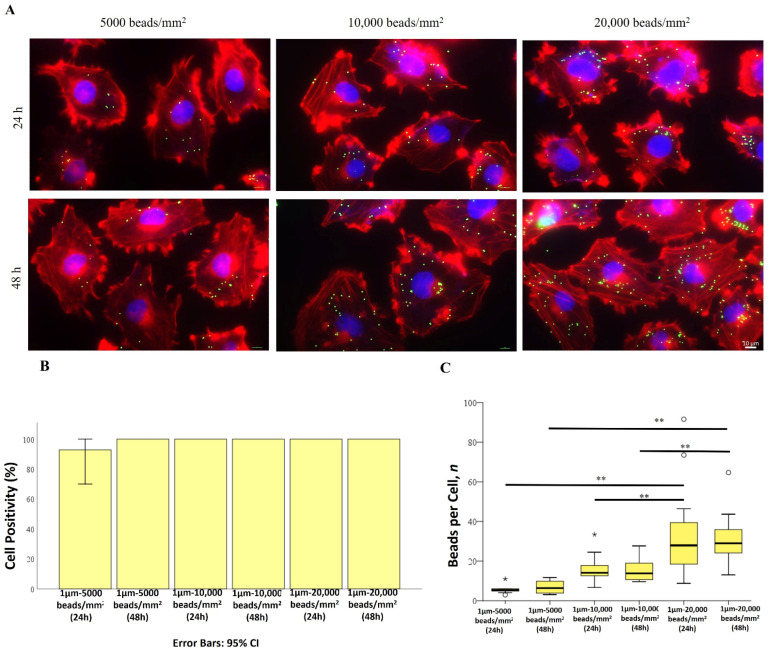
(**A**) Bioaccumulation of 1 µm PS-MPs by Mahlavu cells after 24 h and 48 h of exposure to beads at different densities (5000–10,000–20,000 beads/mm^2^). Fluorescent microscopy representative images of PS-MPs-treated cells. Nucleus: blue (DAPI); cytoskeleton: red (Phalloidin Alexa Fluor-555 conjugated); PS-MPs: green/yellow. Scale bar: 10 µm. (**B**) Percentage of positive cells for PS beads internalization. Error bars: 95% CI. (**C**) number of 1 µm PS-MPs per single cell. * *p* < 0.05; ** *p* < 0.01; circle sign for data normalization.

This study aimed at investigating the impact of increasing the number and exposure time of beads on cells. Bead exposure varied from 5000 to 20,000 beads and from 24 to 48 h (Table S2). Figure 4A illustrates a direct correlation between bead density and bioaccumulation after 24 h of exposure. However, this relationship seems to change when doubling the exposure time to 48 h. To explore this further, bead quantification was performed (Figure 4B,C), revealing that although all cells were positive for beads at both time points, the number of beads per cell differed significantly. Notably, the mean number of internalized beads per cell increased with higher bead densities: 6, 16, and 33 beads per cell for treatments with 5000, 10,000, and 20,000 beads/mm², respectively. This suggests that intracellular internalization may correspond only with the increase in bead density administration.

**Figure 5:**
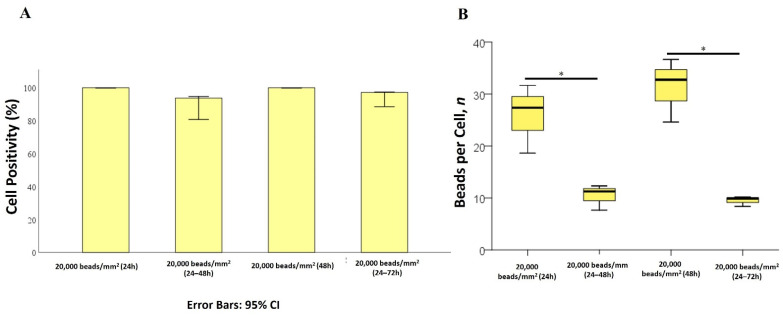
Extrusion process of 1 µm PS-MPs in Mahlavu cells exposed to 20,000 beads/mm^2^ for 24 or 48 h. (**A**) Percentage of positive cells for PS beads internalization. Error bars: 95% CI. (**B**) Number of beads per cell in the different tested conditions. * *p* < 0.05.

To understand whether cells might extrude plastic beads, cells were exposed to flPS-MPs at a density of 20,000 beads/mm^2^ for 24 or 48 h, followed by plastic removal for the next 24 h (24 + 24 or 48 + 24 h). The percentage of PS-MPs-positive cells remained close to 100% in all the tested conditions (Figure 5A), whereas a reduction of more than 60% of internalized PS-MPs was observed. No differences in beads per cell were detected in the 24 or 48 h exposed samples, as they decreased in both cases with an average of nine beads per cell 24 h after beads removal (Figure 5B).

### 2.3. Internalization of 2 µm PS-MPs by Mahlavu Cells

**Figure 6:**
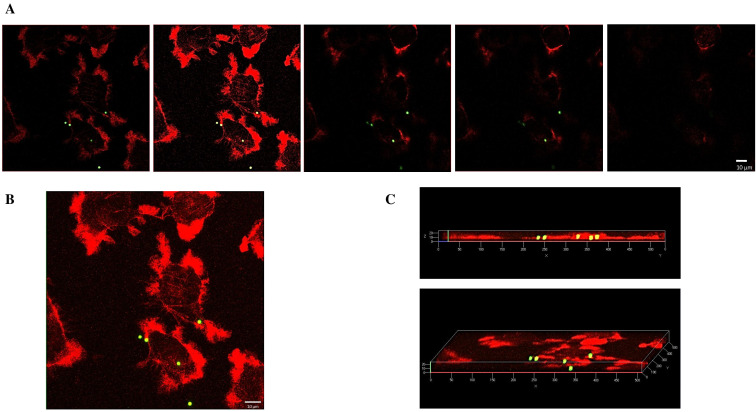
Confocal microscopy of Mahlavu cells after 24 h of exposure to 2 µm PS beads (5000 beads/mm^2^). (**A**) Five consecutive optical sections, Z-step: 0.3 µm; (**B**) Z-stack projection deriving from the superimposition of 26 optical sections with a 0.3 µm Z-step; (**C**) three-dimensional views of the Z-stack along the x-axis (upper image) and the z-axis (lower image). Cytoskeleton: red (Phalloidin Alexa Fluor-555 conjugated); PS-MPs: green/yellow. Scale bar: 10 µm.

Figure 6A shows the internalization of 2 µm beads after 24 h of exposure to 5000 beads/mm^2^, analyzed through confocal microscopy. Both Z-stack projection (Figure 6B) and 3D rendering view (Figure 6C) illustrate bead localization within the cells, with fewer internalized beads observed compared to the 1 µm samples. 

**Figure 7:**
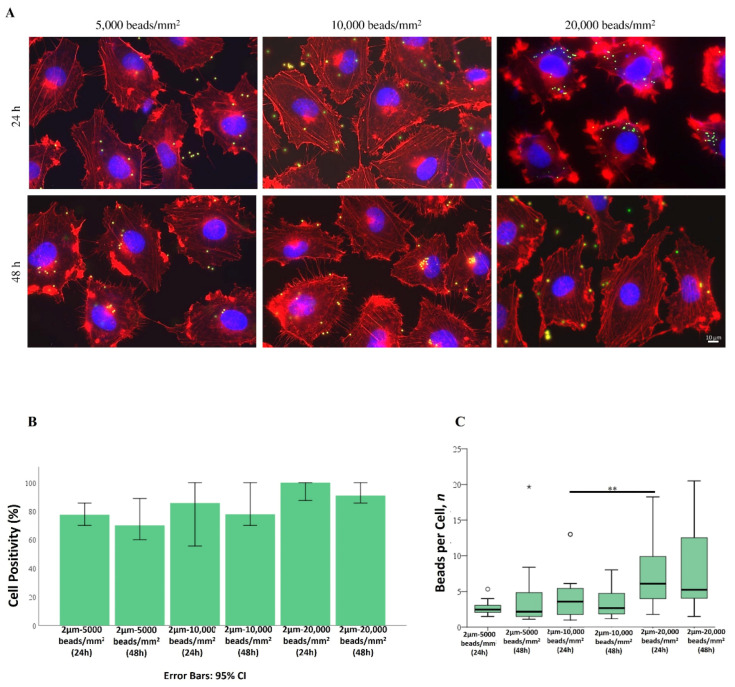
Bioaccumulation and localization pattern of 2 µm PS-MPs in Mahlavu cells, after 24 h and 48 h of exposure to beads at different densities (5000–10,000–20,000 beads/mm^2^). (**A**) Fluorescent microscopy representative images of PS-MPs-treated cells. Nucleus: blue (DAPI); cytoskeleton: red (Phalloidin Alexa Fluor-555 conjugated); PS-MPs: green/yellow. Scale bar: 10 µm. (**B**) Percentage of positive cells for PS beads internalization. Error bars: 95% CI. (**C**) Number of beads per single cell. * *p* < 0.05; ** *p* < 0.01; circle sign for data normalization.

Figure 7A shows the internalization of 2 µm PS-MPs in Mahlavu cells, treated under the same conditions (bead densities and exposure times) as previously described. However, positivity percentages did not reach 100%, even at the highest concentrations and longest exposure times, ranging from 77% in the 5000 beads/mm^2^ treated cells to 83% and 90% in the 10,000 and 20,000 beads/mm^2^ samples, respectively (Figure 7B), with similar values for both exposure times. 

Moreover, compared to 1 µm beads, even the mean number of beads per cell dropped down to four (Figure 7B). Additionally, no significant differences in beads per cell were found among 2 µm-treated samples when comparing exposure time and bead density (Figure 7C).

**Figure 8:**
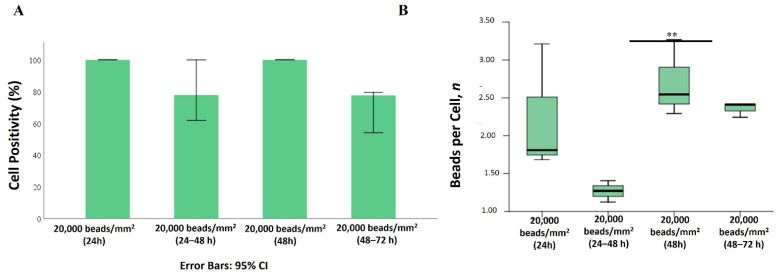
Extrusion of 2 µm PS-MPs. (**A**) Percentage of positive cells for PS beads internalization. Error bars: 95% CI. (**B**) Number of beads per single cell. ** *p <* 0.01.

Despite a decreased bioaccumulation rate, the localization pattern for 2 µm beads was confirmed (peripheral and juxtanuclear localization). Moreover, as a consequence of the PS-MPs removal, contrary to the 1 µm beads, Mahlavu cells seemed unable to extrude 2 µm beads from the cytoplasm. The mean number of 2 µm beads remained almost unchanged at both 24 + 24 and 48 + 24 h (Figure 8).

### 2.4. Internalization of PS-MPs in Human Colon HCT-116 and Lung A549 Cell Lines

**Figure 9:**
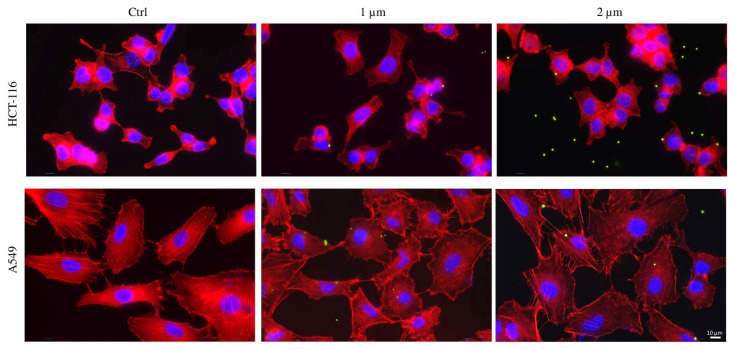
Internalization of 1 and 2 µm flPS-MPs by human cell lines (20,000 beads/mm^2^; exposure time: 48 h). Nucleus: blue (DAPI); cytoskeleton: red (Phalloidin Alexa Fluor-555 conjugated); PS-MPs: green/yellow. Scale bar: 10 µm.

This research aimed to gain insights into plastic bead bioaccumulation by studying HCT-116 and A549 cell lines. Both cell lines were treated for 48 h with 20,000/mm^2^ of 1 or 2 µm PS-MP beads (Supplementary Material, Table S2). Comparing all three cell lines, HCT-116 and A549 exhibited a significantly reduced bioaccumulation rate, with no notable differences observed between bead diameters and the two cell lines. Moreover, the localization pattern differed from what was observed in Mahlavu cells (Figure 9).

### 2.5. Effects of PS-MPs on Cell Viability and Proliferation 

**Figure 10:**
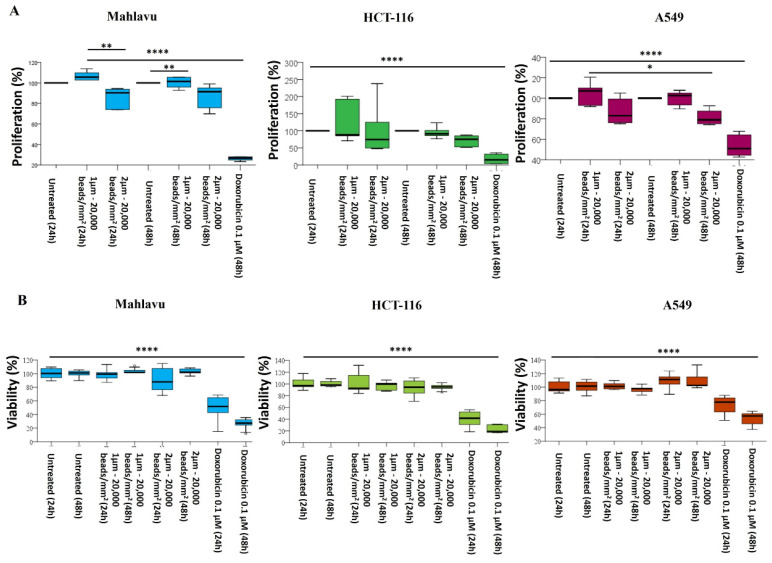
Effects of 1 and 2 µm PS-MPs on cell viability and proliferation of Mahlavu, HCT-116, and A549 cell lines exposed to 20,000 beads/mm^2^. (**A**) Percentage of cell proliferation in cells exposed to PS-MPs for 24 or 48 h. Doxorubicin was used as positive control; (**B**) percentage of cell viability in cells exposed to PS-MPs for 24 or 48 h. Doxorubicin was used as positive control. * *p* < 0.05; ** *p* < 0.01; **** *p* < 0.0001.

The study also aimed at assessing whether PS-MPs could affect cellular fitness through their bioaccumulation, focusing on cell viability and proliferation. Mahlavu, HCT-116, and A549 cell lines were exposed to 1 and 2 µm ulPS-MPs at a density of 20,000 beads/mm^2^ for 24 and 48 h (Supplementary Material, Tables S3 and S4). Since no effect on cell viability was observed in any of the three cell lines (Figure 10A), the exposure time was extended to 72 h. For the 1 µm PS-MPs, no effects on the three cell lines were observed at any examined time. However, different results were noted in the 2 µm experiments (Figure 10B):

(i) Mahlavu and A549 cells exhibited a 20% reduction in proliferation after 24 h, remaining relatively stable for Mahlavu cells up to 72 h, while A549 cells further decreased their proliferation rate to 30%.

(ii) In contrast, HCT-116 cell proliferation remained unchanged for the first 24 h and then decreased by 25% at both 48 and 72 h of bead exposure.

As no differences in cellular viability and proliferation were detected after bead exposure, plastic bead removal experiments were not conducted in HCT-116 and A549 cells.

## 3. Discussion

To better contextualize the results obtained, this section is divided into different sub-paragraphs.

### 3.1. Premise

The widespread presence of MPs in various aspects of daily life, such as food items (sugar, honey, salt) and cosmetics, underscores their ubiquitous nature [39,40]. Additionally, plastic debris, including acrylic, polyester, and nylon, has been identified in lung biopsies from textile industry workers [41]. MPs can enter the human body through inhalation and ingestion and may be transported to other organs via the bloodstream and lymphatic system [39,42,43]. Intestinal absorption of ingested plastic debris can lead to delivery to the liver via the portal vein, highlighting the liver as a target for plastic accumulation and physiological alterations [43,44,45,46,47,48]. MPs-derived liver inflammation has been demonstrated both in vitro and in vivo models, indicating potential hepatotoxic effects [42,44,45,49]. To better understand the impact of specific MPs on human health, if ingested or inhaled, in our in vitro study, we investigated bioaccumulation of PS-MPs in three human cell lines: Mahlavu liver cancer cells, HCT-116 colorectal carcinoma cells, and A549 lung carcinoma cells. Moreover, our experimental plan consisted of the following:

(i) Cell exposition to smaller particles (1 and 2 µm PS-MPs), at more reliable densities (Supplementary Material, Table S2) [50,51], to better simulate environmental plastic pollution intake by humans. The size choice depends on the fact that most studies have been conducted on particles above 10 or 50 µm [36], whereas less data have been reported about smaller particles, such as those described in seawater [52]. The main trouble in studying smaller MPs is given by the actual techniques proposed for sizing and analyzing them. To our knowledge, the most frequently used techniques to identify plastic particles’ chemical composition are micro-Raman spectroscopy and micro-FTIR spectroscopy. However, these techniques have a limited applicability, because of their resolution power (1 and 20 µm for the two methods, respectively) [53,54]. Our aim was therefore to mimic environmental plastic pollution as much as possible, although remaining in a standardized system of in vitro study. Not only MP sizes but even the concentrations were chosen to simulate real plastic contamination. For example, our highest concentration for the 2 µm PS beads (i.e., 20,000 beads/mm^2^) corresponds to 42.29 µg of plastic/mL, which is much lesser than other quantities utilized in other research (Supplementary Material, Table S1), such as 500 µg/mL used by Hwang J. or 1.5 mg/mL used by Zhang Y.X. for their in vitro studies [31,55,56].

(ii) A specific choice of MPs, i.e., PS-MPs. In fact, PS is one of the major contributing pollutants in environmental samples [40,57]; it is hydrophobic yet easily suspended in aqueous media, and its size does not require functionalization to increase permeability through cell membranes [40,58,59]. In addition, PS-MPs can be easily created with precise sizes for binding other molecules, such as fluorescent dyes [40]. Although we used virgin plastics, they can be comparable to environmentally dispersed PS-MPs, as they typically derive from degradation of plastic polymers [60]. Commercially available PS-MPs are produced by shaping the original raw material with heat treatment, maintaining the same molecule composition as the original particles [60,61,62,63,64].

### 3.2. Mahalavu Cell Line

Also, in accordance with previous studies [39,47,65], our major findings in Mahlavu cells define the following:

(i) A specific bead localization pattern: both 1 and 2 µm plastic particles accumulate in cell cytoplasm, from the periphery to the juxtanuclear region, without entering the nucleus, consistently with observations in human Caco-2 cells [66]. 

(ii) A PS-MPs size dependency, where the major uptake rate was observed with 1 µm beads, although, unlike other studies, no significant alteration of cell morphology was observed [28,39,67]. 

In addition, we also noticed that the 1 µm bioaccumulation occurred solely in a dose-dependent manner without any time dependency. Increasing bead density led to a rise in internalized beads, with comparable numbers per single cell between 24 and 48 h of exposure (6, 16, and 33 beads, respectively, for 5000, 10,000, and 20,000 beads/mm^2^ treatments), suggesting, therefore, that PS-MPs exposure time does not significantly influence the bead internalization process. In contrast, bioaccumulation of 2 µm PS-MPs appeared to be a random phenomenon, not observed in all cells and not related to plastic densities or exposure time. Comparing positivity percentages, 100% of Mahlavu cells internalized 1 µm PS-MPs, while the positivity percentage for 2 µm beads ranged from 77% to 90% at the highest concentration (20,000 beads/mm^2^), with a mean of four beads per single cell. This difference in uptake pattern aligns with the role of particle size in cell accumulation [68]. 

### 3.3. HCT-116 and A549 Cell Lines

HCT-116 and A549 cell lines were treated for 48 h with 20,000/mm^2^ 1 or 2 µm PS-MP beads. Comparing all three cell lines, HCT-116 and A549 showed a strongly reduced bioaccumulation rate, and no relevant differences were observed in both bead diameters and the two cell lines (Figure 9). Consequently, even the localization pattern was not the same when compared to what was seen in Mahlavu cells. Moreover, it was not possible to underline a specific bioaccumulation trend. This was also confirmed by viability and proliferation tests: we reported no reduction in cell viability and only a modest decrease in cell proliferation for both HCT-116 and A549 cells exposed to 2 µm PS-MPs, in accordance with other studies [27,69].

### 3.4. General Discussion

Our study emphasizes different crucial points in the MPs uptake process: 

(i) MP dimensions: Various studies have elucidated the relationship between MP and nanoparticle (NP) dimensions, showing how, depending on cell type, shape, and size, after creating hydrophobic and Van der Waals’ interactions, particles may enter cells via either passive penetration (particles smaller than 500 nm) or active transport, such as endocytosis (particles larger than 500 nm) via the endocytosis mechanism [68,70,71,72,73,74,75]. In fact, studies on different cell lines, both human (lung A549, colon CaCo-2, renal HK-2) and murine (macrophage line J774A.1, rat basophilic leukemia cells), confirmed that the size of particles guides the active internalization process, with endocytosis being the preferential route for particles larger than 500 nm [73,74,75]. Given that, since we utilized 1 µm and 2 µm MP beads in our cell models, it is reasonable to assume that the uptake mechanism may be based on the endocytosis process.

To better confirm the relationship between MPs’ cell accumulation and their dimensions, we also observed MPs extrusion processes, for 1 and 2 µm plastics. After removing uninternalized beads, the number of accumulated 1 µm PS-MPs decreased by up to 60% in Mahlavu cells, whereas 2 µm PS-MPs remained inside the cytoplasm, proving that the extrusion process is also dependent on particle dimensions. This observation aligns with findings suggesting that the extrusion process may be hindered by the cell’s inability to degrade plastic particles, leading them to accumulate primarily in lysosomes and remain inside cells [37,67]. Some studies have demonstrated that energy-dependent exocytosis could be the primary mechanism for cell clearance, involving lysosomal pathways and inhibition of retrograde intracellular transport [42,67,76]. Our findings, therefore, confirm that smaller particles are easier for cells to accumulate. 

(ii) Cell area. The more surface cells have, the more they can accumulate. Comparing Mahlavu cells to A549 and HCT-116 cell lines, a different PS-MPs uptake was observed, related to their diverse typology, dimensions, and function in the organism, as also reported by da Silva Brito et al. [77]. Morphologically speaking, Mahlavu cells have larger dimensions, and our findings indicate that the major PS beads accumulation rate is related to that (as shown in Figure 9). 

(iii) Cell physiology and function. Our experimental plan is based on three different epithelial cell lines, which act as barriers or filters. We did not find a consistent reduction in cell proliferation or cell viability in any of them. Liver cells work to protect human organisms from environmental chemicals [78], similar to how pulmonary and intestinal epithelial cells prevent the entry of external molecules [79,80]. Moreover, the few effects on Mahlavu cell viability confirm that the extrusion process helps in maintaining cell viability, above all considering the role of liver cells in protecting the organism from foreign molecules and/or filtering the blood. 

Indeed, and similarly to other studies, no reduction in cell viability was evaluated for HCT-116 and A549 [18,69]. We reported only a modest decrease in cell proliferation for both HCT-116 and A549 cells when exposed to 2 µm PS-MPs. 

(iv) Dose and time exposure. Based on all previous points and considering that only in the Mahlavu cell line was it possible to describe a bioaccumulation trend, we can state that the MPs uptake is only dose- and not time-dependent, making, therefore, a correlation between cell surface, MPs dimensions, and exposure density. 

Moreover, another important parameter to keep under consideration is the cell surface, as we observed that cells with a wider surface allow an easier uptake compared to smaller ones. Lastly, epithelial cell viability and proliferation are not influenced by MPs uptake, or they are only influenced to a small extent. Altogether, our findings create a new step forward for better comprehending the effects of MPs on human health. Knowing these correlations, future studies, for instance, could focus on particles smaller or equal to 1 µm, and it would be possible to predict the fate of MPs in specific human body districts.

## 4. Materials and Methods

### 4.1. PS-MPs

flPS-MP, sized 1 and 2 µm (product number: L9654 and L9529, respectively—Ex. ≈ 500 nm; Em. ≈ 540 nm), and ulPS-MPs, sized 1 and 2 µm (product number: 89904 and 78452, respectively), were purchased from Sigma-Aldrich (St. Louis, MO, USA) in the form of dispersions in distilled H_2_O (10% solid content). Prior to each cellular experiment, PS-MPs stocks were sterilized by ultraviolet (UV) treatment for 20 min, washed with phosphate-buffered saline (PBS) to remove the cytotoxic components of the bead dispersions (e.g., detergent), and diluted in complete cell medium at the required concentrations.

### 4.2. MPs Characterization

For both analyses, beads were diluted 1:100 in distilled H_2_O (ddH_2_O) and spotted on slides. After drying, they were covered by a 10 nm gold layer for SEM analysis (EVO 40, Zeiss; Oberkochen, Germany), whereas the flPS-MPs were fixed with 4% paraformaldehyde (Sigma-Aldrich; St. Louis, MO, USA) in PBS [PFA-PBS] for 30 min at room temperature (RT) and washed 3 times with PBS for 5 min at RT and once with ddH_2_O for 2 min at RT. Afterwards, the slides were mounted with a mounting slide medium (2.3% 1,4-diazabicyclo[2.2.2]octane—DABCO; Sigma-Aldrich; St. Louis, MO, USA—in PBS/glycerol 1:1) and imaged with a Nikon DS-Qi2 fluorescent microscope (Nikon; Tokyo, Japan).

### 4.3. Cell Culture and Treatment

Mahlavu cell line was kindly provided by Dr. Rengul Cetin-Atalay (Bilkent University, Ankara, Turkey) [81,82]. A549 and HCT-116 cell lines were kindly provided by Prof. Paola Secchiero (University of Ferrara, Ferrara, Italy). All of them were maintained in Dulbecco’s modified Eagle’s High Glucose Medium (DMEM) without sodium pyruvate (Lonza; Basel, Switzerland) and supplemented with 10% inactivated fetal bovine serum (FBS) (Sigma-Aldrich; St. Louis, MO, USA), 1% Penicillin/Streptomycin (Lonza; Basel, Switzerland), and 1% L-Glutamine (Lonza; Basel, Switzerland) at 37 °C and 5% CO_2_.

The day before exposure to plastic beads (D−1), cells were plated at different densities, according to their different growth and treatment times and dimensions (Supplementary Materials, Table S1). On D0, cells were exposed to different concentrations of flPS-MPs or ulPS-MPs (5000–10,000–20,000 beads/mm^2^ of well plate) for 24 (D1) or 48 h (D2). Cells that received no plastic treatment were used as negative control, plated at the same density as the respective treated cell line, and allowed to grow for the same amounts of time (24 h and 48 h). 

All the experiments were conducted in triplicate.

### 4.4. Fluorescent Assay

#### 4.4.1. Fluorescent and Confocal Microscopy

To analyze the internalization of PS-MPs and determine the intracellular localization of plastic particles, flPS-MPs-exposed cells were observed by fluorescent microscopy. On D1 and D2, cells were washed to remove non-internalized MPs. After fixation (4% PFA-PBS for 30 min at RT) and cellular permeabilization (0.1% Triton X-100 in PBS for 15 min at RT), cells were stained with Phalloidin Alexa Fluor-555 diluted at 1:2000 in 1% bovine serum albumin (BSA) in PBS for 1 h at RT to label actin filaments. Cell nuclei were stained with 0.05 µg/mL 4′,6-diamidino-2-phenylindole (DAPI) for 30 s at RT. After cell dehydration, with an ascending ethanol series and mounting with 2.3% DABCO in PBS/glycerol (1:1), they were imaged by a Nikon DS-Qi2 fluorescent microscope. Unless specified, images were taken with a Nikon Plan-Apochromatic 60×/1.40 objective immersed in oil.

The fluorescence detection was carried out using DAPI (Ex 340-380; DM 400; BA435-485), FITC (Ex 465-495; DM 505; BA 515-555), and TRITC (Ex 542/20; DM 570; BA 620/52) filters. This study investigated the uptake of PS-MPs at the single-cell level; therefore, we proceeded in counting beads within the cells. However, automatic segmentation and object recognition were not feasible due to the significant overlap between the long emission tail from plastic beads and the emission of phalloidin at 555 nm. Therefore, manual bead counting was performed on 35 cells from 4 randomly selected fields of view (FoVs). An air Plan Fluor 40×/0.75 (Nikon; Tokyo, Japan) objective was used to ensure homogeneous cell scenarios and wider FoVs. Imaging conditions were standardized by keeping acquisition parameters constant for both control and treated cells, including exposure time and gain. Only beads within the cell cytoplasm were counted.

Moreover, to investigate bead release, after cell washing in PBS to remove non-internalized beads, cells were allowed to grow for an additional 24 h in complete cell culture medium without PS-MPs. After fluorescent staining, cells were imaged and bead counted, as previously described. Unwashed cells were used as control. 

Confocal microscopy was employed to confirm PS beads internalization and revealed their distribution within cells. Images were captured using a Zeiss LSM 510 laser scanning confocal microscope (Zeiss; Oberkochen, Germany) with a Plan-Apochromat 63×/1.4 immersion oil objective (Type F, R.I. 1.518 at 23 °C, purchased by Zeiss, Oberkochen, Germany) and 488 and 543 nm lasers for fluorescence detection. 

#### 4.4.2. Image Post-Processing

Image post-processing was performed using ImageJ, Fiji software (open-source image processing based on ImageJ 1.53a software [83]) and ZEN 2009 software (Zeiss; Oberkochen, Germany).

### 4.5. Cell Proliferation Assay

Cell proliferation was assessed using the xCelligence Real-time Cell Analyzer-Dual Plate (Agilent; Santa Clara, CA, USA). On D1, cells were plated in duplicate in E-plate 16 (Roche; Mannheim, Germany) at a density of 1.5 × 10^3^ cells, following the conditions outlined in Supplementary Materials, Table S3. On D0, cells were treated with fresh medium containing 20,000 ulPS-MPs/mm^2^. The assay was performed according to the manufacturer’s instructions. Unexposed and 0.1 µM Doxorubicin-treated cells served as negative and positive controls, respectively. Cell proliferation was continuously monitored in real time for 48 h. The proliferation values for each PS-MPs-treated group at different time points (D1 and D2) were calculated as percentages compared to the unexposed controls. All the experiments were conducted in triplicate.

### 4.6. Cell Viability Assay

To assess the impact of PS-MPs on cellular viability, Cell Counting Kit-8 assay was performed (CCK8; Dojindo Molecular Technologies; Rockville, MD, USA). On D−1, cells were plated in triplicate in 96-well culture plates at varying densities (Supplementary Materials, Table S4). Cellular densities were adjusted to ensure optimal conditions for viability assessment. On the subsequent day, cells were treated with 100 µL of fresh medium containing 20,000 ulPS-MPs/mm^2^ of well plate. Doxorubicin-treated and unexposed cells served as positive and negative cytotoxicity controls, respectively. After 24 and 48 h of PS-MPs exposure, CCK8 reagent was added following the manufacturer’s instructions, and the resulting optical densities (ODs) were measured at 450 nm (measurement filter) and 620 nm (reference filter) using a microplate reader (Infinite M-plex, Tecan, Hombrechtikon, Switzerland). Cell viability values for each PS-MPs-treated group were calculated as percentages relative to the unexposed controls after subtracting the reference OD from the measurement OD. 

### 4.7. Statistical Analysis

The experiments were conducted in triplicate, and statistical analysis was performed using SPSS 28.0 software (IBM SPSS Statistics, IBM Corporation; Armonk, NY, USA). The normality of distribution for continuous variables was assessed using the Shapiro–Wilk test. For normally distributed variables, mean and standard deviation (SD) were used, while non-normally distributed variables were represented by the median and 95% confidence intervals (95% CIs). Categorical data were expressed as total numbers and percentages (%). Percentages were compared using the chi-squared test, while continuous data were compared using Student’s t-test or Mann–Whitney test, as appropriate. Box plots were used to compare beads per cell under different conditions, including different concentrations of beads (5000, 10,000, and 20,000 beads/mm^2^), exposure times, wash-out times, and bead sizes (1 and 2 µm). To assess changes from baseline in cell positivity, the baseline value was set at 100%, and the value after 48 h wash-out (24 h–48 h) represented the change from baseline. A *p*-value < 0.05 was considered statistically significant.

## 5. Conclusions

To our knowledge, this is the first study that has analyzed plastic particles kinetics at the single-cell level, investigating both PS-MPs bioaccumulation and the following extrusion by cells. We assessed the correlation between MPs’ densities and sizes, cell dimensions and functions, and time dependency. MPs size plays a pivotal role in plastic kinetics, since the smallest MPs are more easily internalized and released. Considering only MPs’ size, we underlined that only 1 µm particles show a density dependency and only in hepatic cells. Moreover, we also noticed that PS-MPs extrusion occurred after plastic particles removal from cell medium, figuring a potential defense mechanism from the cells. Regarding the plastic densities tested in this study (comparable to environmental plastic pollution), no significant effects were observed on the cell viability of human hepatic cancer, lung, and colon cancer cell lines; proliferation of both human lung and colon cancer cells was modestly disrupted, although these two cell lines bioaccumulated lower amounts of PS-MPs. Altogether, these results could help with better understanding the potential size-related risks that MPs have on human health. In the future, it could be possible to better circumscribe the MPs exposure risk, in terms of particle size (and therefore density) and tissue sensitivity. Although the radical and total abolition of plastic seems a very hard step to reach, investigating the correlation between MPs size and the bioaccumulation in certain human districts might be useful to limit human plastic toxicity and reduce the exposure to potential plastic sources.

## Data Availability

The original contributions presented in this study are included in the article/supplementary material; further inquiries can be directed to the corresponding author.

## Supplementary Materials

The following supporting information can be downloaded at: https://www.mdpi.com/article/10.3390/ijms252011101/s1.

## References

[1] Conti I., Simioni C., Varano G., Brenna C., Costanzi E., Neri L.M. (2021). Legislation to limit the environmental plastic and microplastic pollution and their influence on human exposure. Environ. Pollut..

[2] Lee Y., Cho J., Sohn J., Kim C. (2023). Health Effects of Microplastic Exposures: Current Issues and Perspectives in South Korea. Yonsei Med. J..

[3] Hollman P. Microplastics and Nanoplastics in Food—An Emerging Issue. www.efsa.europa.eu/en/news/microplastics-and-nanoplastics-food-emerging-issue.

[4] Sridharan S., Kumar M., Bolan N.S., Singh L., Kumar S., Kumar R., You S. (2021). Are microplastics destabilizing the global network of terrestrial and aquatic ecosystem services?. Environ. Res..

[5] Mkuye R., Gong S., Zhao L., Masanja F., Ndandala C., Bubelwa E., Yang C., Deng Y. (2022). Effects of microplastics on physiological performance of marine bivalves, potential impacts, and enlightening the future based on a comparative study. Sci. Total Environ..

[6] Biswas T., Pal S.C. (2024). Emerging threats of microplastics on marine environment: A critical review of toxicity measurement, policy practice gap and future research direction. J. Clean. Prod..

[7] Hidalgo-Ruz V., Gutow L., Thompson R.C., Thiel M. (2012). Microplastics in the Marine Environment: A Review of the Methods Used for Identification and Quantification. Environ. Sci. Technol..

[8] Galafassi S., Nizzetto L., Volta P. (2019). Plastic sources: A survey across scientific and grey literature for their inventory and relative contribution to microplastics pollution in natural environments, with an emphasis on surface water. Sci. Total Environ..

[9] Courtney A., Baker J., Bamford H. Proceedings of the International Research Workshop on the Occurrence, Effects, and Fate of Microplastic Marine Debris.

[10] Atlas E., Dimitrova V. (2019). Bisphenol S and Bisphenol A disrupt morphogenesis of MCF-12A human mammary epithelial cells. Sci. Rep..

[11] Hahladakis J.N., Velis C.A., Weber R., Iacovidou E., Purnell P. (2018). An overview of chemical additives present in plastics: Migration, release, fate and environmental impact during their use, disposal and recycling. J. Hazard. Mater..

[12] de Sá L.C., Luís L.G., Guilhermino L. (2015). Effects of microplastics on juveniles of the common goby (*Pomatoschistus microps*): Confusion with prey, reduction of the predatory performance and efficiency, and possible influence of developmental conditions. Environ. Pollut..

[13] Mistri M., Sfriso A.A., Casoni E., Nicoli M., Vaccaro C., Munari C. (2022). Microplastic accumulation in commercial fish from the Adriatic Sea. Mar. Pollut. Bull..

[14] Zuccarello P., Ferrante M., Cristaldi A., Copat C., Grasso A., Sangregorio D., Fiore M., Conti G.O. (2019). Exposure to microplastics (<10 µm) associated to plastic bottles mineral water consumption: The first quantitative study. Water Res..

[15] Habibi N., Uddin S., Fowler S., Behbehani M. (2022). Microplastics in the atmosphere: A review. J. Environ. Expo. Assess..

[16] Eales J., Bethel A., Galloway T., Hopkinson P., Morrissey K., Short R.E., Garside R. (2022). Human health impacts of exposure to phthalate plasticizers: An overview of reviews. Environ. Int..

[17] Hamilton B.M., Baak J.E., Vorkamp K., Hammer S., Granberg M., Herzke D., Provencher J.F. (2022). Plastics as a carrier of chemical additives to the Arctic: Possibilities for strategic monitoring across the circumpolar North. Arct. Sci..

[18] Li J., Zhang K., Zhang H. (2018). Adsorption of antibiotics on microplastics. Environ. Pollut..

[19] Wright S.L., Kelly F.J. (2017). Plastic and Human Health: A Micro Issue?. Environ. Sci. Technol..

[20] Abbasi S. (2021). Routes of human exposure to micro(nano)plastics. Curr. Opin. Toxicol..

[21] Abbasi S., Turner A. (2021). Human exposure to microplastics: A study in Iran. J. Hazard. Mater..

[22] Schwabl P., Köppel S., Königshofer P., Bucsics T., Trauner M., Reiberger T., Liebmann B. (2019). Detection of Various Microplastics in Human Stool. Ann. Intern. Med..

[23] Jenner L.C., Rotchell J.M., Bennett R.T., Cowen M., Tentzeris V., Sadofsky L.R. (2022). Detection of microplastics in human lung tissue using µFTIR spectroscopy. Sci. Total Environ..

[24] Ragusa A., Svelato A., Santacroce C., Catalano P., Notarstefano V., Carnevali O., Papa F., Rongioletti M.C.A., Baiocco F., Draghi S. (2021). Plasticenta: First evidence of microplastics in human placenta. Environ. Int..

[25] Schirinzi G.F., Pérez-Pomeda I., Sanchís J., Rossini C., Farré M., Barceló D. (2017). Cytotoxic effects of commonly used nanomaterials and microplastics on cerebral and epithelial human cells. Environ. Res..

[26] Forte M., Iachetta G., Tussellino M., Carotenuto R., Prisco M., De Falco M., Laforgia V., Valiante S. (2016). Polystyrene nanoparticles internalization in human gastric adenocarcinoma cells. Toxicol. In Vitro.

[27] Zhang Y., Wang S., Olga V., Xue Y., Lv S., Diao X., Zhang Y., Han Q., Zhou H. (2022). The potential effects of microplastic pollution on human digestive tract cells. Chemosphere.

[28] Di Dong C., Chen C.W., Chen Y.C., Chen H.H., Lee J.S., Lin C.H. (2020). Polystyrene microplastic particles: In vitro pulmonary toxicity assessment. J. Hazard. Mater..

[29] Chiu H.-W., Xia T., Lee Y.-H., Chen C.-W., Tsai J.-C., Wang Y.-J. (2015). Cationic polystyrene nanospheres induce autophagic cell death through the induction of endoplasmic reticulum stress. Nanoscale.

[30] Abbasi S., Moore F., Keshavarzi B. (2021). PET-microplastics as a vector for polycyclic aromatic hydrocarbons in a simulated plant rhizosphere zone. Environ. Technol. Innov..

[31] Jin Y., Lu L., Tu W., Luo T., Fu Z. (2019). Impacts of polystyrene microplastic on the gut barrier, microbiota and metabolism of mice. Sci. Total Environ..

[32] Vandenberg L.N., Luthi D., Quinerly D. (2017). Plastic bodies in a plastic world: Multi-disciplinary approaches to study endocrine disrupting chemicals. J. Clean. Prod..

[33] Deng Y., Zhang Y., Lemos B., Ren H. (2017). Tissue accumulation of microplastics in mice and biomarker responses suggest widespread health risks of exposure. Sci. Rep..

[34] Choi Y.J., Park J.W., Lim Y., Seo S., Hwang D.Y. (2021). In vivo impact assessment of orally administered polystyrene nanoplastics: Biodistribution, toxicity, and inflammatory response in mice. Nanotoxicology.

[35] Domenech J., de Britto M., Velázquez A., Pastor S., Hernández A., Marcos R., Cortés C. (2021). Long-Term Effects of Polystyrene Nanoplastics in Human Intestinal Caco-2 Cells. Biomolecules.

[36] Leslie H.A., van Velzen M.J.M., Brandsma S.H., Vethaak A.D., Garcia-Vallejo J.J., Lamoree M.H. (2022). Discovery and quantification of plastic particle pollution in human blood. Environ. Int..

[37] Chen X., Li X., Li Y. (2021). Toxicity inhibition strategy of microplastics to aquatic organisms through molecular docking, molecular dynamics simulation and molecular modification. Ecotoxicol. Environ. Saf..

[38] Hollóczki O., Gehrke S. (2020). Can Nanoplastics Alter Cell Membranes?. Chem. Phys. chem..

[39] Goodman K.E., Hare J.T., Khamis Z.I., Hua T., Sang Q.-X.A. (2021). Exposure of Human Lung Cells to Polystyrene Microplastics Significantly Retards Cell Proliferation and Triggers Morphological Changes. Chem. Res. Toxicol..

[40] Paul M.B., Stock V., Cara-Carmona J., Lisicki E., Shopova S., Fessard V., Braeuning A., Sieg H., Böhmert L. (2020). Micro- and nanoplastics—Current state of knowledge with the focus on oral uptake and toxicity. Nanoscale Adv..

[41] Warheit D.B., Hart G.A., Hesterberg T.W., Collins J.J., Dyer W.M., Swaen G.M.H., Castranova V., Soiefer A.I., Kennedy G.L. (2001). Potential Pulmonary Effects of Man-Made Organic Fiber (MMOF) Dusts. Crit. Rev. Toxicol..

[42] Yang Y.F., Chen C.Y., Lu T.H., Liao C.M. (2019). Toxicity-based toxicokinetic/toxicodynamic assessment for bioaccumulation of polystyrene microplastics in mice. J. Hazard. Mater..

[43] Lu Y.Y., Li H., Ren H., Zhang X., Huang F., Zhang D., Huang Q., Zhang X. (2022). Size-dependent effects of polystyrene nanoplastics on autophagy response in human umbilical vein endothelial cells. J. Hazard. Mater..

[44] Yong C.Q.Y., Valiyaveettil S., Tang B.L. (2020). Toxicity of Microplastics and Nanoplastics in Mammalian Systems. Int. J. Environ. Res. Public Health.

[45] Shen R., Yang K., Cheng X., Guo C., Xing X., Sun H., Liu D., Liu X., Wang D. (2022). Accumulation of polystyrene microplastics induces liver fibrosis by activating cGAS/STING pathway. Environ. Pollut..

[46] Ge Y., Yang S., Zhang T., Wan X., Zhu Y., Yang F., Yin L., Pu Y., Liang G. (2023). The hepatotoxicity assessment of micro/nanoplastics: A preliminary study to apply the adverse outcome pathways. Sci. Total Environ..

[47] Xu H., Wang J., Wang Q., Tu W., Jin Y. (2024). Co-exposure to polystyrene microplastics and cypermethrin enhanced the effects on hepatic phospholipid metabolism and gut microbes in adult zebrafish. J. Hazard. Mater..

[48] Horvatits T., Tamminga M., Liu B., Sebode M., Carambia A., Fischer L., Püschel K., Huber S., Fischer E.K. (2022). Microplastics detected in cirrhotic liver tissue. eBioMedicine.

[49] Cheng W., Li X., Zhou Y., Yu H., Xie Y., Guo H., Wang H., Li Y., Feng Y., Wang Y. (2022). Polystyrene microplastics induce hepatotoxicity and disrupt lipid metabolism in the liver organoids. Sci. Total Environ..

[50] Cox K.D., Covernton G.A., Davies H.L., Dower J.F., Juanes F., Dudas S.E. (2019). Human Consumption of Microplastics. Environ. Sci. Technol..

[51] Nor N.H.M., Kooi M., Diepens N.J., Koelmans A.A. (2021). Lifetime Accumulation of Microplastic in Children and Adults. Environ. Sci. Technol..

[52] Ter Halle A., Jeanneau L., Martignac M., Jardé E., Pedrono B., Brach L., Gigault J. (2017). Nanoplastic in the North Atlantic Subtropical Gyre. Environ. Sci. Technol..

[53] Schwaferts C., Sogne V., Welz R., Meier F., Klein T., Niessner R., Elsner M., Ivleva N.P. (2020). Nanoplastic Analysis by Online Coupling of Raman Microscopy and Field-Flow Fractionation Enabled by Optical Tweezers. Anal. Chem..

[54] Oßmann B.E., Sarau G., Schmitt S.W., Holtmannspötter H., Christiansen S.H., Dicke W. (2017). Development of an optimal filter substrate for the identification of small microplastic particles in food by micro-Raman spectroscopy. Anal. Bioanal. Chem..

[55] Hwang J., Choi D., Han S., Jung S.Y., Choi J., Hong J. (2020). Potential toxicity of polystyrene microplastic particles. Sci. Rep..

[56] Zhang Y.X., Wang M., Yang L., Pan K., Miao A.J. (2022). Bioaccumulation of differently-sized polystyrene nanoplastics by human lung and intestine cells. J. Hazard. Mater..

[57] Warrillow S., Fisher C., Bellomo R. (2020). Correction and Control of Hyperammonemia in Acute Liver Failure: The Impact of Continuous Renal Replacement Timing, Intensity, and Duration. Crit. Care Med..

[58] Poon C. (2022). Measuring the density and viscosity of culture media for optimized computational fluid dynamics analysis of in vitro devices. J. Mech. Behav. Biomed. Mater..

[59] Rius-Ayra O., Biserova-Tahchieva A., LLorca-Isern N. (2021). Surface-functionalised materials for microplastic removal. Mar. Pollut. Bull..

[60] Gallo F., Fossi C., Weber R., Santillo D., Sousa J., Ingram I., Nadal A., Romano D. (2018). Marine litter plastics and microplastics and their toxic chemicals components: The need for urgent preventive measures. Environ. Sci. Eur..

[61] Sutkar P.R., Gadewar R.D., Dhulap V.P. (2023). Recent trends in degradation of microplastics in the environment: A state-of-the-art review. J. Hazard. Mater. Adv..

[62] Xiang P., Zhang T., Wu Q., Li Q. (2023). Systematic Review of Degradation Processes for Microplastics: Progress and Prospects. Sustainability.

[63] Dauvergne P. (2018). The power of environmental norms: Marine plastic pollution and the politics of microbeads. Environ. Politics.

[64] Arpia A.A., Chen W.H., Ubando A.T., Naqvi S.R., Culaba A.B. (2021). Microplastic degradation as a sustainable concurrent approach for producing biofuel and obliterating hazardous environmental effects: A state-of-the-art review. J. Hazard. Mater..

[65] Wu B., Wu X., Liu S., Wang Z., Chen L. (2019). Size-dependent effects of polystyrene microplastics on cytotoxicity and efflux pump inhibition in human Caco-2 cells. Chemosphere.

[66] Wang Q., Bai J., Ning B., Fan L., Sun T., Fang Y., Wu J., Li S., Duan C., Zhang Y. (2020). Effects of bisphenol A and nanoscale and microscale polystyrene plastic exposure on particle uptake and toxicity in human Caco-2 cells. Chemosphere.

[67] Han S.W., Ryu K.Y. (2022). Increased clearance of non-biodegradable polystyrene nanoplastics by exocytosis through inhibition of retrograde intracellular transport. J. Hazard. Mater..

[68] Thompson R.C., Olsen Y., Mitchell R.P., Davis A., Rowland S.J., John A.W., McGonigle D., Russell A.E. (2004). Lost at Sea: Where Is All the Plastic?. Science.

[69] Hwang J., Choi D., Han S., Choi J., Hong J. (2019). An assessment of the toxicity of polypropylene microplastics in human derived cells. Sci. Total Environ..

[70] Zhao J., Stenzel M.H. (2018). Entry of nanoparticles into cells: The importance of nanoparticle properties. Polym. Chem..

[71] Lesniak A., Salvati A., Santos-Martinez M.J., Radomski M.W., Dawson K.A., Åberg C. (2013). Nanoparticle Adhesion to the Cell Membrane and Its Effect on Nanoparticle Uptake Efficiency. J. Am. Chem. Soc..

[72] Wang J., Cong J., Wu J., Chen Y., Fan H., Wang X., Duan Z., Wang L. (2023). Nanoplastic-protein corona interactions and their biological effects: A review of recent advances and trends. TrAC Trends Anal. Chem..

[73] Rejman J., Oberle V., Zuhorn I.S., Hoekstra D. (2004). Size-dependent internalization of particles via the pathways of clathrin- and caveolae-mediated endocytosis. Biochem. J..

[74] Li Y., Sun Y., Li J., Tang R., Miu Y., Ma X. (2021). Research on the Influence of Microplastics on Marine Life. IOP Conf. Ser. Earth Environ. Sci..

[75] Kuhn D.A., Vanhecke D., Michen B., Blank F., Gehr P., Petri-Fink A., Rothen-Rutishauser B. (2014). Different endocytotic uptake mechanisms for nanoparticles in epithelial cells and macrophages. Beilstein J. Nanotechnol..

[76] Liu L., Xu K., Zhang B., Ye Y., Zhang Q., Jiang W. (2021). Cellular internalization and release of polystyrene microplastics and nanoplastics. Sci. Total Environ..

[77] da Silva Brito W.A., Singer D., Miebach L., Saadati F., Wende K., Schmidt A., Bekeschus S. (2023). Comprehensive in vitro polymer type, concentration, and size correlation analysis to microplastic toxicity and inflammation. Sci. Total Environ..

[78] Grant D.M. (1991). Detoxification pathways in the liver. J. Inherit. Metab. Dis..

[79] Viggiano D., Ianiro G., Vanella G., Bibbò S., Bruno G., Simeone G., Mele G. (2015). Gut barrier in health and disease: Focus on childhood. Eur. Rev. Med. Pharmacol. Sci..

[80] Di Tommaso N., Gasbarrini A., Ponziani F.R. (2021). Intestinal Barrier in Human Health and Disease. Int. J. Environ. Res. Public Health.

[81] Çıkla-Süzgün P., Kaushik-Basu N., Basu A., Arora P., Talele T.T., Durmaz I., Çetin-Atalay R., Küçükgüzel Ş.G. (2015). Anti-cancer and anti-hepatitis C virus NS5B polymerase activity of etodolac 1,2,4-triazoles. J. Enzym. Inhib. Med. Chem..

[82] Tuncbilek M., Guven E.B., Onder T., Cetin Atalay R. (2012). Synthesis of Novel 6-(4-Substituted piperazine-1-yl)-9-(β-d-ribofuranosyl)purine Derivatives, Which Lead to Senescence-Induced Cell Death in Liver Cancer Cells. J. Med. Chem..

[83] Schindelin J., Arganda-Carreras I., Frise E., Kaynig V., Longair M., Pietzsch T., Preibisch S., Rueden C., Saalfeld S., Schmid B. (2012). Fiji: An open-source platform for biological-image analysis. Nat. Methods.

