# Supplementary material for: Bioaccumulation Rate of Non-Biodegradable Polystyrene Microplastics in Human Epithelial Cell Lines
Source: Int J Mol Sci. 2024 Oct 16;25(20):11101. doi: 10.3390/ijms252011101 (PMC11508641; doi:10.3390/ijms252011101)
Supplement: Supplementary file 1 [file ijms-25-11101-s001.zip › ijms-3239357-supplementary.pdf]

Supplementary Materials

Manuscript title

Bioaccumulation Rate of Non-Biodegradable Polystyrene Microplastics in Human Epithelial Cell Lines

Ilaria Conti [1,†], Cinzia Brenna [1,2,†], Angelina Passaro [1] and Luca Maria Neri [1,2,*]

[1] Department of Translational Medicine, University of Ferrara, 44121 Ferrara, Italy; ilaria.conti@unife.it (I.C.); cinzia.brenna@unife.it (C.B.); angelina.passaro@unife.it (A.P.)

[2] Laboratory for Technologies of Advanced Therapies "LTTA"—Electron Microscopy Center, University of Ferrara, 44121 Ferrara, Italy

* Correspondence: luca.neri@unife.it; Tel.: +39-0532-455940.

† These authors contributed equally to this work.

Table S1

Conversion of the number of PS-MPs per well (mm^2) to µg of polystyrene.

**1 µm PS-MPs**	**n. particles / mm²**	**mm² of well**	**n. particles / well**	**µg beads / well**	**µg beads / mL**
	5,000	32	1.60×10^5	0.09	0.04
		962	4.81×10^6	2.64	1.32
	10,000	32	3.2×10^5	0.18	0.09
		962	9.62×10^6	5.29	2.64
	20,000	32	6.40×10^5	0.35	0.18
		962	19.24×10^6	10.57	5.29

**2 µm PS-MPs**	**n. particles / mm²**	**mm² of well**	**n. particles / well**	**µg beads / well**	**µg beads / mL**
	5,000	32	1.60×10^5	0.70	0.35
		962	4.81×10^6	21.14	10.57
	10,000	32	3.2×10^5	1.41	0.70
		962	9.62×10^6	42.29	21.14
	20,000	32	6.40×10^5	2.81	1.41
		962	19.24×10^6	84.58	42.29

PS density: 1.05 g/cm^3; well area for a 96-wells plate: 32 mm^2; well area for a 6-wells plate: 962 mm^2

Table S2

Cellular densities described for cell line and PS-MPS treatment time

Cell line	Plastic treatment (h)	
	24	48
	Density (cells/well)	
Mahlavu	5×10^4	2.5×10^4
HCT-116	2×10^5	1×10^5
A549	1×10^5	5×10^4

Table S3

Cellular densities used for cell line and PS-MPS treatment time for cell proliferation assay (the assay was performed only after 24h of plastic exposure and evaluated after 24 and 48h)

Cell Line	Density (cell/well)
Mahlavu	$1{,}5 \times 10^3$
HCT-116	3×10^3
A549	2×10^3

Table S4

Cellular densities used for cell line and PS-MPS treatment time for viability assay

Cell line	Plastic treatment (h)	
	24	48
	Density (cells/well)	
Mahlavu	3×10^3	$1{,}5 \times 10^3$
HCT-116	5×10^3	3×10^3
A549	4×10^3	3×10^3
